# Treatment outcomes and predictors of success for multidrug resistant tuberculosis MDR TB in Ugandan regional referral hospitals

**DOI:** 10.1038/s41598-025-97027-x

**Published:** 2025-04-23

**Authors:** Godfrey Barteka, Denis Bwayo, Joseph K. B. Matovu, Benon Wanume, Jimmy Patrick Alunyo, Raymond Sseguya, John Peter Masaba, John Stephen Obbo

**Affiliations:** 1 Department of Community and Public Health, Busitema University, Mbale, Uganda; 2 Mbale Regional Referral Hospital, Mbale, Uganda; 3 Department of Research, Mbale Clinical Research Institute, Mbale, Uganda

**Keywords:** Multidrug-resistant tuberculosis, Treatment outcomes, Predictors, Uganda, Infectious diseases, Tuberculosis, Diseases, Health care

## Abstract

Multidrug-resistant tuberculosis (MDR-TB) is a form of tuberculosis caused by strains resistant to both isoniazid and rifampicin, the most critical first-line drugs. Managing MDR-TB presents substantial challenges due to prolonged and costly treatment regimens, which are less effective than those for drug-susceptible TB. These difficulties are further exacerbated in low-resource settings by inadequate healthcare infrastructure, limited diagnostic capacity, and suboptimal access to treatment. Uganda, a high-burden TB country, faces persistent challenges in meeting national MDR-TB treatment targets, with high mortality rates and unfavourable outcomes. This study evaluated the treatment outcomes and factors associated with success among MDR-TB patients in regional referral hospitals. Of the 293 registered patients, 284 were included in the analysis, with a median age of 38 years (IQR: 30–45) and a predominance of male patients (65.1%). Overall, 68.7% of patients achieved successful treatment outcomes, while 31.3% experienced unfavourable outcomes. Multivariate analysis identified weight at treatment initiation (41–49 kg) as significantly associated with poor outcomes. These findings highlight a treatment success rate below national targets, with persistent high mortality and treatment failure in several regions. Addressing these challenges requires the development of innovative therapies and personalized care strategies to improve MDR-TB management in Uganda.

## Introduction

Multidrug-resistant tuberculosis (MDR-TB) is a serious form of tuberculosis caused by strains resistant to isoniazid and rifampicin, the two most effective first-line TB drugs. MDR-TB represents a significant global health threat due to its challenging treatment and high associated mortality^1^. This issue has emerged at a critical time when tuberculosis (TB) control programs, particularly in resource-limited settings, face significant constraints due to the ongoing HIV/AIDS pandemic, chronic underfunding, and shortages of skilled healthcare personnel, especially at lower-level health facilities (Health Centre IIs and IIIs). Additionally, weaknesses in logistics management and inadequate healthcare infrastructure further compromise the effectiveness of TB care and prevention efforts. According to the World Health Organization (WHO), 176,586 cases of MDR-TB were detected in 2022 among bacteriologically confirmed pulmonary TB cases tested for resistance^2^. However, only 63% of patients who started MDR-TB treatment in 2020 achieved successful outcomes, with many unsuccessful cases resulting in death^2^

In response, WHO has introduced measures to improve treatment outcomes for MDR-TB patients, including updated management guidelines and a recommendation to decentralise services WHO, 2019. Studies from sub-Saharan Africa demonstrate that regionalizing MDR-TB services has enhanced patient access to testing, diagnosis, and treatment initiation. Decentralized services have also proven cost-effective in resource-limited settings, achieving higher treatment success rates and improving patient retention in care^3^.

Uganda is one of the 30 high-burden TB/HIV countries, accounting for 90% of the global TB burden^4^. In 2015, the prevalence of MDR-TB among newly diagnosed TB cases in Uganda was estimated at 1.6% (0.78–2.40) and 12% (3.40–21.00) among previously treated cases^5^. According to a 2010 national drug-resistant TB (DR-TB) survey focusing on smear-positive samples reported MDR-TB prevalence rates of 1.4% in new cases and 12.1% in retreatment cases^6^. Sub-national surveys reveal varying MDR-TB prevalence, from 0 to 4.5% in new cases and 4.4–24% in retreatment cases, further underscoring regional disparities. Case retention among drug-susceptible tuberculosis (TB) patients remains a significant challenge. In the 2014/15 cohort, only 79% of new bacteriologically confirmed TB patients achieved treatment success 51% were cured and 28% completed treatment. The remaining 21% experienced unfavourable outcomes, including 5% mortality, 1% treatment failure, 4% unevaluated cases, and 11% lost to follow-up. Treatment outcomes varied across regions; Lira reported the highest success rate (90%), followed by Kampala Capital City Authority (87%) and Masaka (84%), while Moroto (60%), Arua (66%), and Hoima (68%) recorded the lowest rates. Although there were moderate improvements in treatment success between 2008 and 2015, national success rates remained below the 90% target set by the 2015 Stop TB Program, consistently falling under 80%.

This persistently low treatment success rate for MDR-TB highlights gaps that may drive an increase in MDR/RR-TB incidence. Inadequate recording and reporting, lack of definitive treatment outcomes for transferred patients, and challenges in implementing Directly Observed Therapy (DOT) and community-based DOT Short Course Strategy (CB-DOTS) further compound these issues. Limited family and community involvement in patient support and low cure rates suggest potential deficiencies in patient care quality.

Additionally, reports in the country still indicate that treatment outcomes for multidrug-resistant tuberculosis (MDR-TB) patients at tertiary health facilities remain suboptimal, with success rates ranging from 54 to 71.1%^7^. Poor outcomes are driven by patient-related factors, disease severity, inadequate treatment regimens, and gaps in healthcare provider capacity. Additional challenges include substandard ambulatory care, frequent adverse events, and the complexity, cost, and length of treatment regimens, which contribute to poor adherence and high dropout rates^8^. Moreover, the social and psychological needs of MDR-TB patients are often unmet, further complicating treatment^9^. Limited healthcare access, insufficient provider training, and inadequate infrastructure continue to undermine efforts to improve MDR-TB management and align with global TB elimination targets. Given this backdrop, the present study focuses on understanding MDR-TB treatment outcomes and the predictors of poor outcomes to inform targeted improvements in Uganda’s MDR-TB management strategies. Specifically, to address the challenges faced by patients, and improve treatment outcomes at tertiary level health facilities, in resource-constrained settings like Uganda. In this study, we analysed the treatment outcomes and predictors of treatment success among patients managed for multidrug-resistant tuberculosis at selected regional referral hospitals in Uganda.

## Methodology

### Study design

This was a retrospective cohort study conducted at the Mbale, Soroti, Lira, and Moroto regional referral hospitals. The study enrolled all MDR-TB patients who were notified between January 2014 and December 2018. These patients were followed up retrospectively from the time of enrollment until the completion of their treatment. The cohorts were defined by the enrollment dates within the specified period (2014–2018).

### Study setting

This hospital-based study was conducted across four Regional Referral Hospitals (RRHs) in Uganda: Mbale, Soroti, Lira, and Moroto RRHs. These hospitals serve as central hubs for the diagnosis, treatment, and management of MDR-TB cases referred from primary and secondary health facilities within their respective catchment areas. They follow the Uganda National Tuberculosis and Leprosy Programme (NTLP) guidelines, which are aligned with World Health Organization (WHO) standards for MDR-TB diagnosis and treatment.

During the study period (2014–2018), Drug Sensitivity Testing (DST) was used for the diagnosis of MDR-TB, identifying resistance to Rifampicin and Isoniazid. The standard MDR-TB treatment regimen included a combination of second-line anti-TB drugs, administered over a course of 18–24 months. An ambulatory treatment model was primarily followed, with patients receiving care in specialized MDR-TB clinics while residing at home or in community-based settings. This was supplemented by directly observed therapy (DOT) to ensure adherence.

Patient support models involved a range of services, including counseling, social support, and nutritional assistance, aimed at addressing both the physical and psychological challenges of treatment. Healthcare workers at varying levels provided care, including doctors, nurses, and laboratory technicians, with training support provided by medical interns and university staff.

Treatment regimens were adjusted accordingly based on test results. These hospitals play a vital role in the management of MDR-TB by offering advanced diagnostic and treatment services while also contributing to research and training healthcare personnel across Uganda.

### Study population

The study population consisted of bacteriologically or clinically confirmed pulmonary TB cases who had undergone the Drug Sensitivity Test (DST) to confirm the presence of *Mycobacterium Tuberculosis* strains resistant to both Rifampicin and Isoniazid drugs. All patients diagnosed and treated at the selected regional referral hospitals from January 2014 to December 2018 were included.

### Sample size

A complete census of eligible patients over the study period was done because the total number of patients was limited. All patients registered in the selected regional referral hospitals between 2014 and 2018 of both sexes and with different ages were eligible to participate. There were 293 MDR-TB patients registered during this period in the four regional referral hospitals; however, only those with complete records were enrolled in the study (n = 284 patients; 96.9%).

### Sampling technique

The study included all the MDR-TB in the selected Regional Referral Hospitals whose data was collected and recorded in the patient charts between January 2014 and December 2018. A case was included if the treatment outcome was recorded in the patient’s chart.

### Recruitment criteria

#### Inclusion criteria

A patient was included in the study if he/she was enrolled for treatment from 1st January 2014 and up to those who ended treatment on 31^st^ December 2018 and the outcome of the treatment assigned.

#### Exclusion criteria

A patient was excluded from the study if he/she was enrolled for treatment outside the period between 1 January 2014 and 31 December 2018 and those whose treatment outcome was not recorded or the assigned treatment outcome was neither died, cured, treatment completed, defaulted, treatment failed, or lost to follow-up.

### Data management and processing

Data extraction was carried out between September and November 2020 using a pre-designed tool to collect relevant information from the medical records of all MDR-TB patients managed from January 2014 to December 2018. The data extraction form captured sociodemographic details (gender, age, education, employment, marital status, starting weight, and religion) as well as clinical features and MDR-TB treatment outcomes.

Before data collection, all data clerks underwent training on record retrieval and use of the extraction tool. Four data clerks, one for each hospital, were supervised by the Principal Investigator (PI). The extraction forms were pretested to ensure consistency and completeness. Any necessary adjustments to the tool were made prior to the final data collection.

The collected data were imported into an Excel database, which was then exported to STATA 14.2 for cleaning, validation, and analysis. The data were checked for inconsistencies, coding errors, completeness, and missing information. To ensure data integrity, backups were stored on an external hard drive and flash drives, and access to the data was restricted through password protection. All procedures followed the Declaration of Helsinki and relevant ethical guidelines.

### Measurement of variables

#### Outcome variable

In this study, the main outcome variable was treatment success. Other outcomes documented were cure, defaulting, loss to follow-up, and death.

#### Predictor variables

The predictors include patient characteristics (age, sex, marital status, education level, occupation, and housing condition) clinical characteristics (TB history, HIV co-infection, History of hospitalization, Sputum conversion, and Haematological parameters) and treatment phase (intensive phase and Continuous phase).

### Data analysis plan

Data were coded and entered in an Excel sheet. It was then transferred to a statistical software STATA version 14.2 for analysis. Categorical data were summarized as frequencies and percentages. Continuous data were summarized as mean and standard deviation. Statistical significance was considered at 0.05 level of significance. Determinants of successful treatment outcomes and unsuccessful treatment outcomes were determined, using the chi-square test. Logistic regression was used for multivariate analysis to identify determinants that were independently associated with the treatment outcomes.

## Results

### Participants recruitments

A total of 293 MDR-TB patient records from four regional referral hospitals were reviewed for the study. After excluding nine records due to incomplete data, 284 patient records were included in the final analysis. Of these, 195 patients achieved successful treatment outcomes, either being cured or completing treatment, while 89 experienced unsuccessful outcomes, including loss to follow-up, treatment default, or death.

### Patient characteristics

Table 1 shows the patient characteristics. The mean age of the 284 patients was 38.2 years with a standard deviation of 14.1 years. More than fifty percent (53.5%) were patients aged 36 years and above. A majority (73.2%) of patients were from a rural setting. Regarding sex, 65% were male. Slightly more than forty percent (42%) had attained a secondary and higher level of education. Nearly two-thirds (63%) were married. Slightly below fifty percent (47%) of patients were working in agricultural enterprises. A majority (56%) of the patients were from Lira Regional Referral Hospital. Nearly sixty percent (59%) of the patients were on retreatment. About 54% were positively living with HIV. Slight less than fifty percent (48%) of the patients weighed between 41 and 49 kg at the initiation of treatment in a given regional referral hospital, with a standard deviation of 9.8 units. See Table 1.

**Table 1:** Distribution of patient and clinical characteristics.

Patient characteristics	Frequency n (284)	Percentage % (100)
Age		
< 25	42	14.8
26–35	90	31.7
36+	152	53.5
Mean ± SD		**38.2 ± 14.1**
Residence		
Rural	208	73.2
Urban	76	26.8
Sex		
Male	185	65.1
Female	99	34.9
Education		
None	59	20.8
Primary	107	37.7
Secondary & Higher	118	41.6
Marital status		
Not married	58	20.4
Married	178	62.7
Other	48	16.9
Religion		
Catholic	95	33.5
Protestants	67	24.6
Other	122	43.0
Occupation		
None	86	30.3
Agric. Enterprise	134	47.2
Professional	10	3.5
Other	54	19.0
Treatment RRH		
Lira	159	56.0
Mbale	84	29.6
Moroto	17	6.0
Soroti	24	8.5
Year of admission		
2014	40	14.1
2015	58	20.4
2016	112	39.4
2017	55	19.4
2018	19	6.7
Clinical characteristics		
Type of patients		
New	116.0	40.9
Retreatment	168	59.1
HIV co-infection		
Yes	132.0	46.5
No	152	53.5
Weight (Kgs)		
< 40	31	10.9
41–49	136	47.9
50+	101	41.2

Significant values are in bold.

### Treatment outcomes of MDR-TB

Table 2 below shows the treatment outcomes of MDR-TB patients during their treatment periods in the study sites. About 69% of the patients treated in the four Regional Referral Hospitals were characterised as cured. There was 1.8% treatment failure. About 12% of the patients were lost to follow up and nearly one in five (18%) of the patients died. See Table 2.

**Table 2:** Characteristics of MDR-TB treatment outcomes.

Variables	Frequency n (284)	Percentages% (100)
Treatment outcomes		
Cured	195	68.7
Treatment failure	5	1.8
Lost to follow up	34	12.0
Dead	50	17.6

### MDR-TB treatment outcomes by study site

Table 3 below shows that the cure rates for Moroto RRH and Soroti RRH are 88.2% and 79.2% respectively. The death rates for Lira RRH and Mbale RRH are 18.9% and 19.1% respectively. Moroto RRH recorded no treatment failure or death.

**Table 3:** MDR-TB treatment outcomes by study site.

Variable	Regional referral hospital				Total n = 284 (100.0)
	Lira, n (%) = 159 (100.0)	Mbale, n (%) = 84 (100.0)	Moroto, n (%) = 17 (100.0)	Soroti, n (%) = 24 (100.0)	
Cured	107 (67.3)	54 (64.3)	15 (88.2)	19 (79.2)	195 (68.7)
Treatment failure	1 (0.63)	4 (4.8)	0 (0.0)	0 (0.0)	5 (01.8)
Lost to follow up	21 (13.2)	10 (11.9)	2 (11.76)	1 (4.2)	34 (12.0)
Dead	30 (18.9)	16 (19.1)	0 (0.0)	04 (16.7)	50 (17.6)

### Predictors of treatment outcomes of MDR-TB patients

#### Association between predictors and treatment outcomes

Table 4 illustrates the association between predictors and treatment outcomes. Among the factors examined at Bivariate, notable differences in treatment success rates emerged across age groups, HIV co-infection status, and weight categories, with each showing significant results.

**Table 4:** Association between predictors and treatment outcomes.

Socio-demographic characteristics	MDR-TB treatment outcome		Total	*p*-value
	Successfully treated	Unsuccessfully treated		
Age				0.019*
< 25	27 (64.3)	15 (35.7)	42 (100.0)	
26–35	56 (62.2)	34 (37.9)	90 (100.0)	
36+	112 (73.7)	40 (26.3)	152 (100.0)	
Residence				0.614
0Rural	147 (70.7)	61 (29.3)	208 (100.0)	
Urban	48 (63.2)	28 (36.8)	76 (100.0)	
Sex				**0.057**
Male	119 (64.3)	66 (35.7)	185 (100.0)	
Female	76 (76.8)	23 (23.2)	99 (100.0)	
Education				0.781
None	39 (66.1)	20 (33.9)	59 (100.0)	
Primary	77 (72.0)	30 (28.0)	107 (100.0)	
Secondary and Higher	79 (67.0)	39 (33.1)	118 (100.0)	
Marital status				0.103
Not married	34 (58.6)	24 (41.4)	58 (100.0)	
Married	127 (71.4)	51 (28.7)	178 (100.0)	
Other	34 (70.8)	14 (29.2)	48 (100.0)	
Religion				0.085
Catholic	66 (69.5)	29 (30.5)	95 (100.0)	
Protestants	53 (79.1)	14 (20.9)	67 (100.0)	
Other	76 (62.3)	46 (37.7)	122 (100.0)	
Occupation				0.156
None	56 (65.1)	30 (34.9)	86 (100.0)	
Agric. Enterprise	98 (73.1)	36 (26.9)	134 (100.0)	
Professional	9 (90.0)	01 (10.0)	10 (100.0)	
Other	32 (59.3)	22 (40.7)	54 (100.0)	
Treatment Hospital				0.189
Lira	107 (52.9)		159 (100.0)	
Mbale	54 (29.7)		84 (100.0)	
Moroto	15 (7.7)		17 (100.0)	
Soroti	19 (9.7)		24 (100.0)	
Treatment year				0.363
2014	28 (70.0)	12 (30.0)	40 (100.0)	
2015	37 (63.8)	21 (36.2)	58 (100.0)	
2016	81 (72.3)	31 (27.7)	112 (100.0)	
2017	38 (69.1)	17 (30.9)	55 (100.0)	
2018	11 (57.9)	08 (42.1)	19 (100.0)	
Clinical characteristics				
Type of patient				0.170
New	85 (73.3)	31 (26.7)	116 (100.0)	
Retreatment	110 (65.5)	58 (34.5)	168 (100.0)	
HIV Co-infection				**0.001**
No	96 (72.7)	36 (27.3)	132 (100.0)	
Yes	99 (65.1)	53 (34.9)	152 (100.0)	
Weight at the initiation of treatment				**0.0001**
< 40	24 (77.4)	07 (22.6)	31 (100.0)	
41–49	77 (56.6)	59 (43.4)	136 (100.0)	
50+	94 (80.3)	23 (19.7)	117 (100.0)	

Significant values are in bold.

Firstly, patients aged 36 years and above achieved a treatment success rate of 73.7%, compared to 62.2% among those aged 26–35 and 64.3% among those under 25. This difference in proportions was statistically significant (*p* = 0.019), indicating that older age may positively influence treatment success.

Secondly, among patients without HIV co-infection, the treatment success rate was 72.7%, higher than the 65.1% success rate seen in co-infected patients. This difference was also significant (*p* = 0.001), suggesting that HIV-negative status is associated with better treatment outcomes.

Lastly, initial weight was a key factor. Patients weighing 50 kg or more had the highest treatment success rate at 80.34%, whereas those in the 41–49 kg range had a success rate of 56.6%, and those under 40 kg had a success rate of 77.4%. This variation in success rates by weight was statistically significant (*p* = 0.000), emphasizing that higher body weight at treatment initiation may be linked to improved MDR-TB outcomes. See Table 4.

### Multivariate analysis

Multivariable logistic regression was conducted to identify factors associated with unsuccessful treatment outcomes, including death, loss to follow-up, and treatment failure while adjusting for potential confounders. Adjusted odds ratios (AOR) with 95% confidence intervals (CI) were reported.

After adjustment, only baseline weight at treatment initiation was significantly associated with treatment outcome. Patients weighing 41–49 kg at treatment initiation had a 67% lower likelihood of treatment success compared to those weighing less than 40 kg (AOR = 0.33, 95% CI 0.12–0.86; *p* = 0.023). No statistically significant associations were observed for age or HIV co-infection. See Table 5.

**Table 5:** Multivariate results for factors associated with MRD-TB treatment success.

	COR (95% CI)	*p*-value	AOR (95% CI)	*p*-value
Age				
< 25	1		1	
26–35	0.92(0.43–1.96)	0.819	0.97(0.42–2-24)	0.941
36+	1.56(0.75–3.22)	0.234	1.78(0.79–3.99)	0.162
HIV co-infection				
No	1		1	
Yes	0.70(0.42–1.16)	0.170	0.82(0.48–1.40)	0.471
Weight at the initiation of treatment				
< 40	1		1	
41–49	0.38(0.15–0.94)	0.037*	0.33(0.12–0.86)	0.023*
50+	1.9(0.46–3.11)	0.719	0.99(0.35–2.78)	0.988

Significant values are in bold.

## Discussions

### Patient and clinical characteristics

In the present study, the mean age ± SD was 38.24 ± 14. The mean age of 38.24 years (± 14 years) observed in this study can be explained by several factors related to the epidemiology and socio-demographic characteristics of MDR-TB in Uganda. For instance, individuals in their late 30 s are typically part of the active workforce, increasing their exposure to TB due to greater social interaction and mobility. Our findings align with those of Ibrahim et al. in Egypt, who reported that the average age of multidrug-resistant tuberculosis (MDR-TB) patients was 39 years. This consistency suggests a similar age distribution of MDR-TB patients across different settings, highlighting the need to focus on this demographic group in TB control programs^10^.

In our study, males accounted for 65% of the MDR-TB cases, while females represented 35%. This aligns with established epidemiological trends, where MDR-TB disproportionately affects men. The higher prevalence among males can be attributed to greater exposure to infection through occupational and social activities that increase their risk of transmission. Conversely, sociocultural and economic barriers may limit healthcare access for women, potentially resulting in underdiagnosis and an artificially lower incidence rate among females. Also, this comes following WHO reports that included more males than females as men account for a high proportion of notified MDR-TB cases^11^. This result is also consistent with the study conducted in Egypt by^10^. which reported that the percentage of MDR-TB males was 75.9% and that of females was 24.1%. The result of the present study is also in agreement with Sagwa and others who reported a similar finding that the percentage of MDR-TB males was 66% and that of females was 24%^11^. A similar result was found in the southeast of Turkey Dal 2017 who reported that men were more than females.

We also found that 33.71% of the patients were unemployed, while 40.45% were involved in agricultural activities such as crop farming and livestock keeping. These forms of manual labour likely contribute to increased physical stress, which may exacerbate vulnerability to MDR-TB. This highlights the potential link between occupational factors and TB risk, emphasising the need for targeted interventions within agricultural communities. This present study was matched with Hamdy and colleagues who stated that the highest incidences of TB infections were found also among manual workers^12^. The current findings were also consistent with those in Egypt by Ibrahim et al.^10^. However, contrary to the current findings, Duarte and others found that 51.8% of MDR-TB cases were among unemployed patients^13^. This may be due to low socio-economic status, ignorance, and illiteracy. Furthermore, this may also be due to illness that causes debilitation of patients making them unable to work.

### MDR-TB treatment outcomes

We also found that the treatment success rate was 69% in the present study. This could be explained by the high loss to the follow-up rate, poor treatment management and poor treatment adherence of patients enrolled on MDR-TB treatment. Our result is however less than the 75% target treatment success rate set by the Global Plan to End TB^14^ and other studies (70–77%)^15^. The treatment success rate in this study was also lower than the 75.9% observed in Pakistan^16^. In the current study, treatment failure was 1.8% which was lower than the findings from other countries like Pakistan at 6.3%^16^, and Uzbekistan 8%^17^. Similarly, there was 1.76% treatment failure. This was lower than those from other countries like Pakistan 6.3%^16^, Uzbekistan 8%^17^.

This study identified a high mortality rate of 18% among MDR-TB patients. Several factors likely contribute to this high mortality, including co-morbidities such as HIV and malnutrition, which are prevalent among TB patients. Additionally, delayed diagnosis, inadequate treatment regimens, poor patient adherence, and limited healthcare access, particularly in rural areas, may further exacerbate disease progression and contribute to the observed mortality. Our finding is consistent with findings from studies in Pakistan^16^. However, this rate is notably lower than the 36% mortality reported in South Africa by Dheda et al.^18^.

### Predictors of treatment success for MDR-TB patients

Several predictors of treatment success for MDR-TB cohorts were tested in univariate and multivariate analyses. weight was found to be associated with poor outcomes.

In this study, unemployment was found to be associated with an increased risk of multidrug-resistant tuberculosis (MDR-TB). This finding suggests that the lack of stable employment, which is closely tied to low income, may contribute to the higher burden of MDR-TB in economically disadvantaged populations. This contrasts with a study conducted in the USA by Khan, which found no significant association between employment status and the occurrence of MDR-TB^19^. However, the association observed in our study aligns with several other reports highlighting the greater vulnerability to MDR-TB among individuals from low socioeconomic backgrounds. Raviglione and Director (2013) noted that lower socioeconomic status is a significant risk factor for MDR-TB, likely due to factors such as limited access to healthcare, poor nutrition, and living conditions that facilitate the spread of TB^20^.

In this study, we found that low body weight at admission was strongly associated with poor treatment outcomes in patients with multidrug-resistant tuberculosis (MDR-TB). Specifically, lower baseline weight was identified as a significant risk factor for adverse treatment outcomes. This finding is consistent with results from several other studies, including those by Hicks et al. in South Africa^21^, Janmeja and others in India^22^, and Podewils et al. in Latvia^23^, all of which reported similar associations between low weight and poor treatment response in MDR-TB patients.

### Strengths and limitations

The study used a retrospective cohort design allowed for the tracking of patient outcomes over a five-year period, which offers valuable insights into the long-term effectiveness of MDR-TB treatments.

The choice to include only complete records with sufficient information helped to mitigate biases that could arise from incomplete data, enhancing the study’s internal validity. Also, Conducting the study in Uganda’s regional referral hospitals, which manage a high volume of MDR-TB cases, increases the study’s relevance for public health policy in areas with similar healthcare access challenges and MDR-TB burden.

However, our study primarily used clinical and demographic data, potentially overlooking socioeconomic factors (e.g., income level, employment stability, access to support networks) that may significantly influence treatment outcomes.

We also excluded patients who transferred to other healthcare facilities, which might have impacted the representativeness of treatment outcomes for patients who changed facilities due to relocation or seeking alternative care.

Our study might have also suffered from a lack of a standardized method for missing data, as missing data were addressed by consulting medical records officers when available; however, this approach may not be uniformly reliable across all cases and could lead to inconsistencies in the dataset.

## Conclusions and recommendations

The study found a successful MDR-TB treatment outcome of 68.66% [C.I 63.00–73.82] in four Regional Referral Hospitals. Despite efforts to eliminate TB by 2030, significant mortality from MDR-TB persists, especially in Eastern, Northern, and North-Eastern Uganda. Social demographic and clinical factors, such as age, sex, residence, occupation, education, and HIV co-infection, were not identified as predictors of treatment outcomes. However, low weight (< 50 kg) emerged as a key factor in unsuccessful treatment outcomes. Focus should be placed on this high-risk group to improve treatment success.

To improve future MDR-TB outcomes in Uganda, more treatment centres should be established in rural areas, along with programs to enhance treatment adherence, such as patient counselling.

Future research could replicate this study across all regional referral hospitals to provide a larger sample size and improve understanding of MDR-TB outcomes.

Additionally, a community-based study would capture a wider range of social and cultural factors influencing outcomes, helping to develop effective intervention strategies.

## Data Availability

The datasets used and/or analyzed during the current study are available from the corresponding author upon reasonable request.

## Abbreviations

AIDS: Acquired immunodeficiency syndrome
AOR: Adjusted odds ratio
BMI: Body mass index
CB-DOT: Community based directly observed therapy
COR: Crude odds ratio
DOT: Directly observed therapy
DR-TB: Drug resistance tuberculosis
DS-TB: Drug susceptibility tuberculosis
FY: Financial year
HBC: High burden countries
HIV: Human immunodeficiency virus
MDGs: Millennium development goals
MDR-TB: Multidrug resistance tuberculosis
MOH: Ministry of health
NDRS: National drug resistance survey
NTDs: Neglected tropical diseases
NTLP: National tuberculosis & leprosy program
PMDT: Programmatic management of drug resistance tuberculosis
PTB: Pulmonary tuberculosis
RRHs: Regional referral hospitals
RR-TB: Rifampicin-resistant tuberculosis
SDGs: Sustainable development goals
SSA: Sub-saharan Africa
TB: Tuberculosis
UN: United Nations
WHO: World health organization
XDR-TB: Extensive drug resistance tuberculosis
